# Melamine Exacerbates Neurotoxicity in D-Galactose-Induced Neuronal SH-SY5Y Cells

**DOI:** 10.1155/2023/6635370

**Published:** 2023-11-23

**Authors:** Juhi Goyal, Preet Jain, Vivek Jain, Dibyajyoti Banerjee, Rajasri Bhattacharyya, Sharmistha Dey, Rambabu Sharma, Nitish Rai

**Affiliations:** ^1^Department of Biotechnology, Mohanlal Sukhadia University, Udaipur, India; ^2^Department of Pharmaceutical Sciences, Mohanlal Sukhadia University, Udaipur, Rajasthan, India; ^3^Department of Experimental Medicine and Biotechnology, Postgraduate Institute of Medical Education and Research (PGIMER), Chandigarh, India; ^4^Department of Biophysics, All India Institute of Medical Sciences (AIIMS), New Delhi, India; ^5^Department of Microbiology, Pacific Institute of Medical Sciences, Udaipur, India

## Abstract

Numerous studies have depicted the role of diet and environmental toxins in aging. Melamine (Mel) is a globally known notorious food adulterant, and its toxicity has been shown in several organs including the brain. However, till now, there are no reports regarding Mel neurotoxicity in aging neurons. So, this study examined the *in vitro* neurotoxicity caused by Mel in the D-galactose (DG)-induced aging model of neuronal SH-SY5Y cells. In the present study, the neuronal SH-SY5Y cells were treated with DG and Mel separately and in combination to assess the neurotoxicity potential using MTT assay and neurite length measurement. Further, the superoxide dismutase (SOD), catalase (CAT), and total antioxidant activities were evaluated followed by the determination of the intracellular reactive oxygen species (ROS), mitochondrial membrane potential (MMP), and caspase3 (Casp3) activity. The cotreatment of Mel and DG in neuronal SH-SY5Y cells showed maximum cell death than the cells treated with DG or Mel individually and untreated control cells. The neurite length shrinkage and ROS production were maximum in the DG and Mel cotreated cells showing exacerbated toxicity of Mel. The activity of SOD, CAT, and total antioxidants was also found to be lowered in the cotreatment group (Mel + DG) than in Mel- or DG-treated and untreated cells. Further, the combined toxicity of Mel and DG also elevated the Casp3 activity more than any other group. This is the first study showing the increased neurotoxic potential of Mel in an aging model of neuronal SH-SY5Y cells which implicates that Mel consumption by the elderly may lead to increased incidences of neurodegeneration like Alzheimer's disease and Parkinson's disease.

## 1. Introduction

Age-related neurodegenerative diseases are a significant public health concern worldwide, and their pathogenesis involves a complex interplay of genetic, environmental, and lifestyle factors. The prevalence of these devastating disorders continues to rise as populations age, making it imperative to explore potential risk factors that may contribute to their pathogenesis [1]. Among the various factors, environmental exposures to chemical toxins have emerged as potential contributors to neurodegenerative diseases [2]. Melamine (Mel), a widely used industrial chemical, has garnered attention due to its widespread presence in consumer products such as dairy and baby food as an adulterant, raising concerns about its potential adverse health effects [3].

Mel (1,3,5-triazine-2,4,6-triamine) is a white synthetic solid and nitrogen-rich compound, produced commercially from urea and is commonly employed in the manufacturing of plastics, resins, and adhesives [4]. It is also extensively used as a component in the production of various kitchenware, food packaging materials, and insecticides [5, 6]. However, the occurrence of Mel in food products as a result of contamination or intentional adulteration has been a subject of scrutiny [7]. Notably, Mel contamination incidents, such as the 2008 Chinese milk scandal, have led to widespread public health crises and highlighted the need for a comprehensive understanding of its potential health impacts [8]. Studies have also reported the presence of Mel in potable water sources from industrial effluents and in foodstuffs from Mel-based tableware [9–11].

While Mel is known to be excreted primarily by the kidneys in humans and animals, concerns have been raised about its potential adverse effects on other organ systems, particularly the central nervous system [12, 13]. Several studies have reported on the renal toxicity of Mel, linking it to the formation of crystals and urinary tract complications. However, the specific impact of Mel on neuronal health, especially in the context of aging, remains relatively understudied. Aging is a complex biological process associated with a gradual decline in physiological functions, including those of the brain. The accumulation of oxidative stress and dysregulation of apoptosis are key contributors to the aging-related degeneration of neuronal cells [14, 15].

Findings have suggested that Mel induces apoptosis in neuronal cells which results in serious damaging effects on the brain [13, 16]. Erisgin reported exposure of Mel (50 mg/kg and 75 mg/kg) during the weaning period in Female Wistar albino rats, causing apoptosis in the developing brain [16]. Han et al. demonstrated Mel as an inducer of apoptosis in the differentiated PC12 cells [17].

One of the distinguishing characteristics of an early marker of apoptosis is the disturbance of active mitochondria which includes changes in the oxidation-reduction potential and modifications in the membrane potential. Apoptosis is mediated by cysteine proteases called caspases among which Casp3 executes proteolytic cleavage and cell death process [14, 15].

Understanding the neurotoxic effects of Mel in the context of aging has far-reaching implications for public health. Given the widespread use and potential for Mel contamination in various consumer products, elucidating its impact on neuronal health can inform risk assessment and preventive measures. Furthermore, as aging populations continue to grow, identifying potential environmental risk factors that may contribute to neurodegenerative diseases becomes even more critical for developing targeted interventions and safeguarding the well-being of vulnerable individuals [18]. Hence, investigating the effects of Mel in an aging model could shed light on its potential neurotoxicity and its implications for neurodegenerative diseases in aging populations. D-Galactose (DG) is a reducing sugar (aldohexose) that exists naturally in the body, including the brain, and in several food items such as milk, honey, cheese, yogurt, and beets [19]. Exogenous treatment of DG induces generation of reactive oxygen species (ROS), apoptosis, inflammation along with elevation of aging markers such as advanced glycation end (AGE) products, receptors for advanced glycation end (RAGE) product, senescence-associated gene expression, and so on across diverse cell types [19, 20]. Previous research has established that DG mimics the natural aging process in humans and is a standard choice for assessing the toxicity and protective effect of various compounds against the progression of aging [21].

While earlier research has linked Mel exposure to adverse effects on various physiological systems, its impact on neuronal aging remains relatively unexplored. This study aims to investigate the neurotoxic effects of Mel exposure in an *in vitro* aging model using SH-SY5Y cells treated with DG. By exposing these cells to varying concentrations of Mel, we investigated its potential to exacerbate neurotoxicity and its underlying adverse physiological effect.

## 2. Materials and Methods

### 2.1. Materials

Ham's F12 Nutrient media was acquired from GIBCO, USA. Fetal bovine serum, MTT, DG, Glucose, DMSO, EZAssay™ ROS Assay Kit (FRAP) (CCK078), EZAssay™ Antioxidant Activity Estimation Kit (CUPRAC) (CCK071), and EZAssay™ MMP Assay Kit with JC-1 (CCK079) were purchased from HiMedia, India. The antibiotic-antimycotic solution was collected from CELL clone, India. 96-well cell culture plate, 35 mm Petri dish, 96-well black cell culture plate with a transparent bottom, and T25 flask were procured from Genetix Biotech, India. Mel was from CDH, India. The CAT (707002) and SOD (706002) assay kits were purchased from Cayman Chemical Company, Michigan. The Casp3 Activity Assay Kit (E-CK-A311) was supplied by Elabscience Biotechnology, USA.

### 2.2. Cell Culture

The SH-SY5Y neuroblastoma cell line was procured from NCCS, Pune, India, and cultured in Ham's F12 Nutrient media enriched with 1% (v/v) antibiotic-antimycotic solution and 10% (v/v) fetal bovine serum at 37°C and 5% CO_2_ under humidified conditions. The cells (authenticated by STR profiling) between the passage number 31–35 were used in the experiment and grown as a monolayer.

### 2.3. Cytotoxicity Assay

The cell cytotoxicity was measured using an MTT assay. The cells were seeded in a 96-well cell culture plate at a density of 1 × 10^4^ cells per well for 24 h and then treated with Mel at different concentrations (0.1, 0.2, 0.4, 0.8, 1.0, 1.5, 2.0, 3.0, and 4.0 mg/ml) for 72 h. In another set of experiment, the cells were treated with DG (200 mM) [21, 22] and Mel (1.5 mg/ml) separately and in combination for 72 h. To rule out the glucotoxicity, glucose (200 mM) treatment was also given in one group of cells. After completion of the treatment time, MTT (5 mg/ml) was added and incubated for a further 2 h at 37°C. Absolute DMSO was then added to each well to dissolve the precipitates and incubated for 45 minutes. The absorbance was recorded using iMARK plate reader (Biorad, USA) at 570 nm [23]. The cell viability was expressed as a percentage relative to the viability of untreated control cells.

### 2.4. Neurite Length Measurement

The neurite length measurement was performed to analyze the cell shrinkage and cell death that may be caused by Mel and DG treatment. For determining neurite length, cells were seeded at a density of 0.3 × 10^6^ cells in a 35 mm Petri dish and treated with DG (200 mM) and Mel (1.5 mg/ml) alone and in combination for 72 h. After incubation, photographs were taken and recorded for each treated group at 20x magnification under an inverted microscope (Radicon Scientific Instruments Co., India). Three fields from each condition were selected, and the length of 10 neurites from each field was measured from the center of the cell body to the extreme end of the neurite using ImageJ software (NIH, USA). In total, the length of 30 neurites per treatment group was measured and the total mean length was calculated [24, 25].

### 2.5. Measurement of Intracellular ROS

The cells were seeded in a 96-well black cell culture plate with a transparent bottom at a density of 1 × 10^4^ cells per well for 24 h, and the treatment was performed as described earlier. After incubation, the reaction was executed strictly according to the manufacturer's protocol (EZAssay™ ROS Assay Kit (FRAP) CCK078).

### 2.6. Total Antioxidant Activity Assay

The antioxidant activity was measured using EZAssay™ Antioxidant Activity Estimation Kit (CUPRAC) (CCK071). The cells were seeded in a 35 mm Petri dish at a density of 0.3 × 10^6^ cells per dish and were incubated for 24 h followed by the treatment as described previously. On completion of the treatment time, cells were harvested using rubber policeman and centrifuged at 1500 × *g* for 10 minutes at 4°C. The supernatant was discarded, and the pellet was dissolved in 1x Phosphate buffered saline (PBS). The cells were homogenized on ice, and the whole homogenate was used for the assay according to the manufacturer's protocol. The untreated cells were taken as control, and Trolox was used as standard (supplied in the kit). The activity was expressed as Trolox equivalents calculated using a linear regression equation obtained from the standard curve.

### 2.7. CAT and SOD Assay

The cells were seeded at a density of 0.3 × 10^6^ cells in a 35 mm Petri dish, and appropriate treatment was given. The cells were harvested by rubber policeman and collected by centrifuging at 1000–2000 × *g* for 10 minutes at 4°C. For CAT assay, the cell pellet was homogenized in an ice-cold 50 mM potassium phosphate buffer (containing 1 mM EDTA, pH 7.0) followed by centrifugation at 10,000 × *g* for 15 minutes at 4°C. The supernatant was stored at −80°C and was used for the assay. Further, the reaction was performed according to the manufacturer's protocol (CAT assay kit 707002). For SOD assay, the pellet was homogenized in cold 20 mM HEPES buffer (pH 7.2) containing 1 mM EGTA, 210 mM mannitol, and 70 mM sucrose, followed by centrifugation at 1500 × *g* for 5 minutes at 4°C. The supernatant was collected for the assay and stored at −80°C. Then, the assay was carried out exactly according to the manufacturer's instructions (SOD assay kit 706002).

### 2.8. MMP (ΔΨm) Assay

The cells were seeded in a 96-well black cell culture plate with a transparent bottom at a density of 1 × 10^4^ cells per well for 24 h followed by the treatment. After incubation, the reaction was performed in accordance with the protocol provided by the manufacturer (EZAssay™ MMP Assay Kit with JC-1 CCK079).

### 2.9. Casp3 Activity Assay

The Casp3 activity was determined using the Casp3 Activity Assay Kit (Elabscience E-CK-A311). The cells were seeded in T25 flask at a density of 0.7 × 10^6^ cells, and treatment was given. After the incubation, cells were collected by trypsinization and centrifuged at 2000 rpm for 5 minutes. The pellet was gently resuspended and washed in PBS and collected by centrifugation for 5 minutes at 2000 rpm. The cell pellet was lysed in cold lysis buffer (supplied in the kit) for 30 minutes. The lysed cells were subjected to centrifugation at 12,000 rpm for 15 minutes at 4°C. The supernatant for the assay was stored at −70°C. The assay was conducted in accordance with the manufacturer's specified methodology.

### 2.10. Statistical Analysis

The results are expressed as mean ± SEM. The statistical analysis was performed using one-way ANOVA with the post hoc Tukey test. The *p* value less than 0.05 was considered statistically significant. At levels of 0.001 (^*∗∗∗*^*p* < 0.001), 0.01 (^*∗∗*^*p* < 0.01), and 0.05 (^*∗*^*p* < 0.05), differences were regarded significant. All the experiments were performed in triplicates.

## 3. Results

### 3.1. Mel-Induced Cytotoxicity in DG-Treated Cells

**Figure 1:**
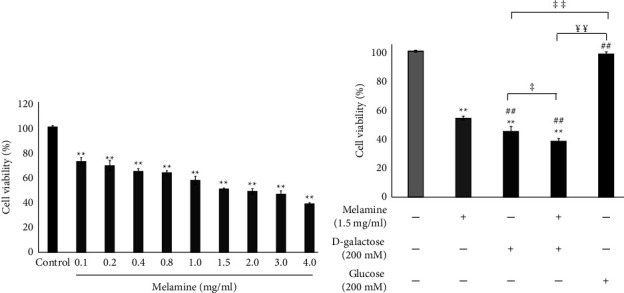
Melamine-induced cell death in a dose-dependent manner. (a) Melamine cytotoxicity. SH-SY5Y cells were treated with different concentrations of melamine for 72 h. Cytotoxicity was assessed through MTT assay. Data are reported as mean ± SEM. Statistically significant differences are indicated as ^*∗∗*^*p* < 0.01 compared to the control group. (b) The effect of Melamine on cell proliferation following D-galactose-induced toxicity. SH-SY5Y cells were treated individually and in combination with melamine and D-galactose for 72 h. Another group of cells was treated only with glucose for 72 h. Cytotoxicity was evaluated using MTT assay. Data are reported as mean ± SEM. Statistically significant differences are indicated as ^*∗∗*^*p* < 0.01 compared to the control group, ^##^*p* < 0.01 compared to the melamine group, ^‡‡^*p* < 0.01 and ^‡^*p* < 0.05 compared to the D-galactose group, and ^¥¥^*p* < 0.01 compared to the glucose group.

The cytotoxicity of Mel individually and in combination with DG was analyzed using MTT assay (Figure 1). The exposure of SH-SY5Y cells to various concentrations of Mel (0.1–4.0 mg/ml) for 72 h showed significant cytotoxicity (*p* < 0.01) in a dose-dependent manner (Figure 1(a)). The IC_50_ was found to be 1.5 mg/ml, where 50% cell death was observed with respect to control. When cells were treated with DG (200 mM), there was an increased cell death than Mel (1.5 mg/ml). Interestingly, when the cells were cotreated with DG (200 mM) and Mel (1.5 mg/ml), a higher cell death was observed than in cells treated with either DG (200 mM) or Mel (1.5 mg/ml) alone (Figure 1(b)) (*p* < 0.01). The cells treated with a comparable dose of glucose (200 mM) exhibited no significant decline in cell viability ruling out glucotoxicity.

### 3.2. Mel Treatment Shortened Neurite Length in DG Exposed Cells

**Figure 2:**
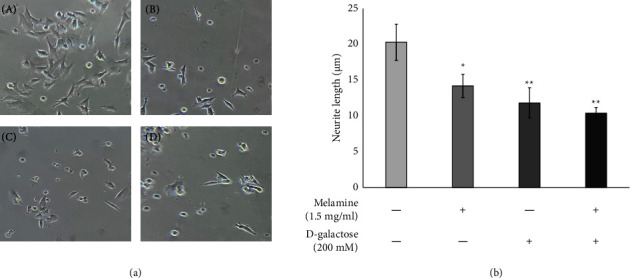
Effect of melamine on the neurite length of the cells following D-galactose-induced aging. (a) Representative pictures for each group (20x magnification). (A) Control, (B) melamine, (C) D-galactose, and (D) melamine + D-galactose. (b) The neurite length of SH-SY5Y cells for each group was measured using ImageJ software. Data are reported as mean ± SEM. Statistically significant differences are indicated as ^*∗∗*^*p* < 0.01 and ^*∗*^*p* < 0.05 compared to the control group.

The treatment of Mel and DG resulted in a significant decline in the neurite length in all the treated groups than in the control groups (Figure 2(a)). However, the cells cotreated with DG and Mel exhibited strong neurite degeneration among all the treatment groups (Figure 2(b)) (*p* < 0.01). Moreover, there was higher neurite shortening in the DG-treated group (*p* < 0.01) than Mel (*p* < 0.05).

### 3.3. Mel and DG Elevated Intracellular ROS

**Figure 3:**
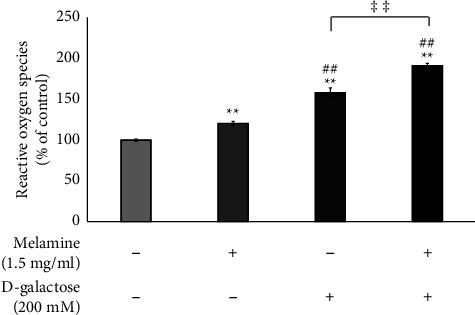
Effect of melamine on reactive oxygen species (ROS) levels in cells following D-galactose-induced aging. Data are reported as mean ± SEM. Statistically significant differences are indicated as ^*∗∗*^*p* < 0.01 compared to the control group, ^##^*p* < 0.01 compared to the melamine group, and ^‡‡^*p* < 0.01 compared to the D-galactose group.

The intracellular ROS levels were significantly elevated in all the treated groups. A higher ROS level was observed in Mel-treated cells (*p* < 0.01) and further raised in DG-treated cells (*p* < 0.01) than in the control group. However, the cotreatment of Mel and DG exhibited maximum ROS levels indicating the highest oxidative stress (Figure 3) (*p* < 0.01).

### 3.4. Mel Attenuated the Antioxidant Activity in DG-Treated SH-SY5Y

**Figure 4:**
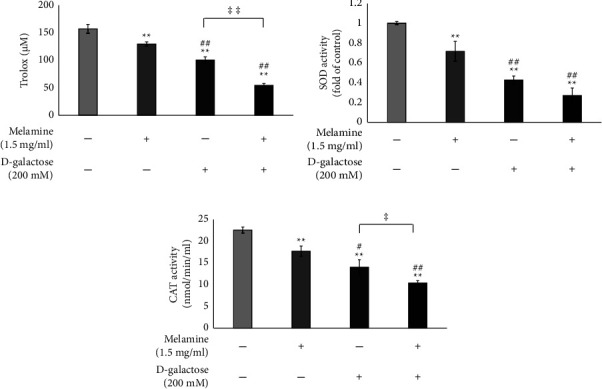
Effect of melamine on (a) total Antioxidant activity, (b) superoxide dismutase (SOD) activity, and (c) catalase (CAT) activity in cells following D-galactose-induced aging. Data are reported as mean ± SEM. Statistically significant differences are indicated as ^*∗∗*^*p* < 0.01 compared to the control group, ^##^*p* < 0.01 compared to the melamine group, ^‡‡^*p* < 0.01, and ^‡^*p* < 0.05 compared to the D-galactose group.

The SH-SY5Y cells treated with Mel exhibited weakened antioxidant activity (*p* < 0.01) which was found to be further attenuated in the case of DG-treated cells (*p* < 0.01). However, the cells cotreated with Mel and DG showed the least antioxidant activity indicating the highest vulnerability to oxidative stress (Figure 4(a)) (*p* < 0.01).

### 3.5. Mel Abrogated SOD and CAT Activity in Aged Cells

The attenuated antioxidant activity of the cells was further evaluated by assessing the activities of SOD and CAT enzymes. The activity of both SOD and CAT enzymes was found to be inhibited in all the treated cells with maximum inhibition in the case of cotreatment with DG and Mel (Figures 4(b) and 4(c)). A 0.7-fold decrease in the activity of SOD was observed in cells cotreated with DG and Mel than in the control group (Figure 4(b)) (*p* < 0.01). In contrast, cells treated with DG and Mel individually exhibited 0.6- and 0.3-fold reduction in SOD activity, respectively (*p* < 0.01). The activity of CAT in cells cotreated with DG and Mel declined by 12 nmol/min/ml with respect to the control (Figure 4(c)) (*p* < 0.01). The cells treated with Mel only and DG only showed a reduction in the CAT activity by 4.8 (*p* < 0.01) and 8.54 nmol/min/ml (*p* < 0.01), respectively.

### 3.6. Mel Decreased MMP in DG-Treated SH-SY5Y

**Figure 5:**
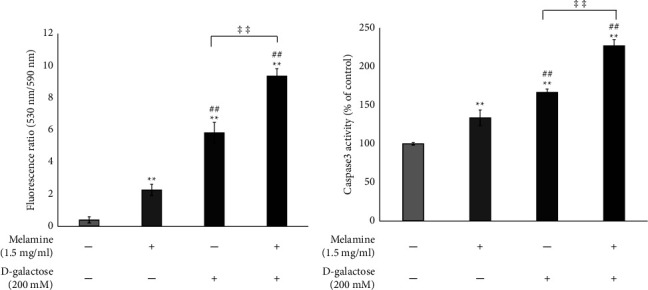
Effect of melamine on (a) mitochondrial membrane potential (MMP) and (b) caspase3 (Casp3) activity following D-galactose-induced aging in cells. Data are reported as mean ± SEM. Statistically significant differences are indicated as ^*∗∗*^*p* < 0.01 compared to the control group, ^##^*p* < 0.01 compared to the melamine group, and ^‡‡^*p* < 0.01 compared to the D-galactose group.

The MMP of all the treated groups was measured as the ratio of fluorescence at 530 nm and 590 nm. The higher the ratio, the lower the MMP. The MMP of control cells was found to be stable but a significant decrease in the MMP was observed in Mel-treated (*p* < 0.01) and DG-treated cells (*p* < 0.01) with maximum decline in the cotreatment group (both Mel and DG) (*p* < 0.01) (Figure 5(a)).

### 3.7. Mel Exposure-Induced Casp3 Activity

The Casp3 activity was induced in all the treated groups; however, the cells cotreated with Mel and DG displayed the highest Casp3 activity than the Mel or DG-treated cells (Figure 5(b)) (*p* < 0.01). Among all the treated groups, Mel-treated cells showed minimum Casp3 activity; however, it was significantly higher than the control group (*p* < 0.01).

## 4. Discussion

Aging is a multifaceted phenomenon that depends on an interplay of numerous factors including environmental exposure to chemical toxins and dietary patterns that may regulate the aging process [26]. Mel has been reported to be present in several pesticides and used as a food adulterant [27, 28]. Owing to its toxic nature, exposure to Mel either directly or indirectly may affect several organs and organ systems including brain [29]. The neurotoxicity of Mel has been shown in some animal studies, indicating cognitive impairment due to oxidative stress-induced synaptic dysfunction in the hippocampus [13, 30–33]. In our study, we investigated the potential effects of Mel on aged neurons using an *in vitro* cellular model of aging induced by DG and elucidated the underlying adverse biochemical effect on cellular physiology. To the best of our knowledge, this study is the first one to report the toxic effects of Mel on aged neuronal cells and demonstrated the increased vulnerability of such cells to Mel exposure.

Mel-induced apoptosis and oxidative damage on PC12 cells have been shown in a previous study where the cells exposed for 36 h exhibited 50% cell viability at 990 *μ*g/ml dose of Mel [34]. In this study, we demonstrated that the treatment of Mel on SH-SY5Y cells for 72 h triggered cell death in a concentration-dependent manner with IC_50_ at 1.5 mg/ml dose (Figure 1(a)). Further, when DG-treated (200 mM) SH-SY5Y cells were exposed to toxic dose of Mel (1.5 mg/ml) for 72 h, a greater reduction in the cell viability was observed than in cells treated with DG or Mel individually (Figure 1(b)). This indicates the elevated toxicity of Mel in presence of DG, enhancing the neuronal cell death.

Further, to assess the effect of Mel in inducing neuronal morphological changes, the neurite length was measured post exposure. The neurite length was found to be significantly decreased in all treatment groups (Figure 2(b)); however, among all the groups, the cotreatment of DG and Mel caused an utmost decline in the neurite length. Comparatively, the effect of Mel without DG on the shortening of neurite length was not so severe. Earlier studies have suggested the inhibitory effects of several environmental toxins on neurite length [35, 36], and it may be possible that Mel treatment in aging cells impaired neurite integrity and led to neurite degeneration [37]. Former reports have depicted a linkage between age-related neurodegenerative disorders such as Alzheimer's disease and Parkinson's disease (PD) and neurite degeneration as it is a primary event that precedes the onset of symptoms [38, 39].

To interrogate the probable mechanism of enhanced neurotoxicity by Mel, we evaluated the intracellular ROS levels in the cells. An elevated intracellular ROS level was found in all treatment groups, with the highest levels in the cells cotreated with the DG and Mel (Figure 3). This finding suggests that although the production of ROS is known in aging [7], it gets further boosted upon treatment with Mel, depicting a rise in oxidative stress. Hence, cotreatment of DG and ML poses a risk of greater deleterious effects at the cellular level.

Oxidative stress is a consequence of an imbalance between ROS production and its subsequent elimination by the antioxidant defense mechanisms [8]. In this line, we evaluated the total antioxidant activity of the cells. Our data showed that Mel treatment on SH-SY5Y neuronal cells attenuated the antioxidant activity causing oxidative stress conditions. This result was in accordance with the previous reports that showed increased oxidative stress in the hippocampal region of adult rats upon treatment with Mel [15]. Further in our study, we found that when DG-treated cells were exposed to Mel, more pronounced decrease in the antioxidant activity of the cells occurred, hence depicting huge oxidative stress in the cotreated group (Figure 4(a)). This suggested that Mel treatment further disrupts the overall oxidation-antioxidation homeostasis of the aged cells.

Furthermore, the activity of antioxidant enzymes such as CAT and SOD was examined as it is evident that a decline in endogenous antioxidants leads to excessive ROS formation [9]. Upon treatment of SH-SY5Y cells with DG and Mel individually, a marked decrease in the activity of SOD and CAT was observed (Figures 4(b) and 4(c)). This is in agreement with the previous studies which showed a decline in the activity of both these enzymes in the DG-induced aging model [10], as well as in cells and animal models treated with Mel [11–13]. However, when cells were cotreated with DG and Mel, a maximum reduction in the activity of both enzymes was found rendering cells more vulnerable to the damaging effects of ROS (Figures 4(b) and 4(c)) [14].

Generation of ROS and oxidative stress has been associated with the activation of caspases which results in apoptosis [16]. One of the characteristics of apoptosis is the loss of MMP. It is an early event that occurs before phosphatidylserine externalization on the plasma membrane and during caspase activation [40, 41]. Caspases are members of the broad protein family among which Casp3 is an effector caspase that cleaves nuclear scaffold and key cellular proteins and activates DNase for degrading nuclear DNA [42]. Therefore, further in line, we evaluated MMP and Casp3 activities to validate apoptosis. A significant alteration in MMP and increased caspase activity were found in all the treatment groups. The fluorescence ratio at 530 nm/590 nm was highest for cells cotreated with Mel and DG than Mel-treated or DG-treated cells which indicated that the cotreatment resulted in greater loss of MMP resulting in maximum apoptosis (Figure 5(a). In an earlier study, increased activity of Casp3 in DG-treated cells is reported and the apoptosis induction in PC12 cells upon exposure to Mel has been shown by Han et al. [17, 18]. Our findings were consistent with the earlier studies and revealed the maximal Casp3 activity in cells cotreated with DG and Mel (Figure 5(b)). Increased Casp3 activity has been recognized as a marker of apoptosis [19]. Hence, the extreme decrease in SH-SY5Y cell viability could be attributed to the apoptosis induced by Mel and DG synergistically.

**Figure 6:**
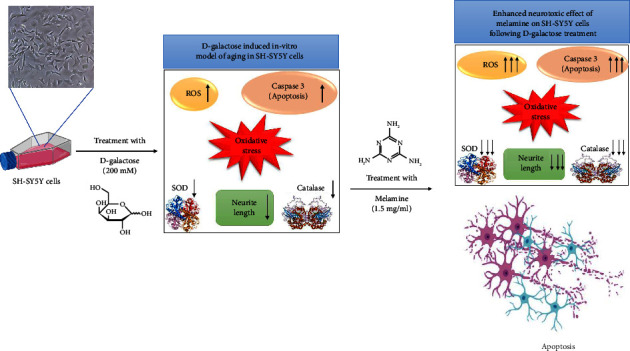
Schematic representation showing the mechanism of melamine toxicity in D-galactose-induced aging in SH-SY5Y cells (Figure created with BioRender.com).

Our study revealed that Mel has serious detrimental effects on aged SH-SY5Y cells generated using D-galactose. Oxidative stress, ROS formation, and apoptosis were found to be enhanced along with the depletion of antioxidants in the Mel-treated aging cellular model (Figure 6). Owing to the ability of Mel to cross the blood-brain barrier, it has also been shown to cause memory and learning deficits via impairing synaptic plasticity and altering neurotransmission [29–32]. Therefore, it is possible that Mel ingestion by the elderly population may lead to detrimental consequences making them more vulnerable to cognitive impairment and age-related neurodegenerative diseases such as Alzheimer's and Parkinson's disease.

## 5. Conclusion

Our study demonstrated the enhanced neurotoxic effect of Mel on an aging model of SH-SY5Y cells. These findings contribute to the growing body of knowledge on diet and environmental factors that may influence neuronal health and pave the way for further research in this area. Ultimately, a comprehensive understanding of the neurotoxic mechanisms of Mel will aid in the formulation of evidence-based public health strategies and regulations to mitigate potential risks associated with Mel exposure in aging populations.

## Data Availability

The experimental data used to support the findings of this study are included within the article.

## Abbreviations

Mel: Melamine
DG: D-Galactose
ROS: Reactive oxygen species
SOD: Superoxide dismutase
CAT: Catalase
MTT: 3-(4,5-Dimethyl-2- thiazolyl)-2,5-diphenyl-2H-tetrazolium bromide
MMP: Mitochondrial membrane potential
PBS: Phosphate buffered saline
Casp3: Caspase3.
